# Target Doppler Rate Estimation Based on the Complex Phase of STFT in Passive Forward Scattering Radar

**DOI:** 10.3390/s19163627

**Published:** 2019-08-20

**Authors:** Karol Abratkiewicz, Piotr Krysik, Zbigniew Gajo, Piotr Samczyński

**Affiliations:** Institute of Electronic Systems, Faculty of Electronics and Information Technology, Warsaw University of Technology, 00-665 Warsaw, Poland

**Keywords:** passive forward scattering radar, chirp rate estimation, passive radar, forward scattering radar, radar measurements, time-frequency analysis

## Abstract

This article presents a novel approach to the estimation of motion parameters of objects in passive forward scattering radars (PFSR). In such systems, most frequency modulated signals which are used have parameters that depend on the geometry of a radar scene and an object’s motion. Worth noting is that in bistatic (or multistatic) radars forward scattering geometry is present thus in this case only Doppler measurements are available while the range measurement is unambiguous. In this article the modulation factor, also called the Doppler rate, was determined based on the chirp rate (equivalent Doppler rate) estimation concept in the time-frequency (TF) domain. This approach utilizes the idea of the complex phase of the short-time Fourier transform (STFT) and its modification known from the literature. Mathematical dependencies were implemented and verified and the simulation results were described. The accuracy of the considered estimators were also verified using the Cramer-Rao lower bound (CRLB) to which simulated data for the considered estimators was compared. The proposed method was validated using a real-life signal collected from a radar operating in PFSR geometry. The Doppler rate provided by a car crossing the baseline between the receiver and the GSM transmitter was estimated. Finally, the concept of using CR estimation, which in the case of PFSR can be understood as Doppler rate, was confirmed on the basis of both simulated and real-life data.

## 1. Introduction

**Figure 1:**
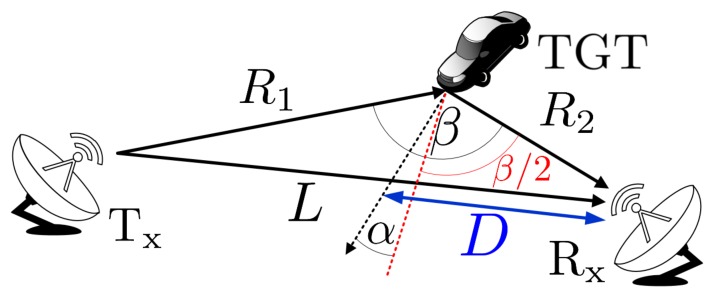
Simplified passive forward scattering radar (PFSR) geometry. β—bistatic angle, $T_{x}$—non-cooperative transmitter, $R_{x}$—radar receiver, TGT—target, *L*—baseline, $R_{1}$—distance from the transmitter to the target, $R_{2}$—distance from the target to the receiver, *D*—distance from the receiver to the crossing point.

Passive forward scattering radars are a special class of passive bistatic radars (PBR), in which the bistatic angle β between the non-cooperating transmitter, the target and the receiver is $\beta \approx 180^{\circ }$ [1,2,3]. In such kinds of passive radars, the illuminating signal can be a wave from a commercial transmitter of popular systems such as FM, DAB, DVB-T, GSM, and so forth [4,5,6,7]. The simplified passive forward scattering radar (PFSR) geometry is presented in Figure 1.

Unlike typical PBRs, the PFSR is characterized by the fact that objects cross the baseline, which has certain consequences. In such a case the PBR radar using classical PCL (Passive Coherent Location) processing is blind, as a target is crossing the line of sight between a receiver and a transmitter, and for PBR in this geometry there is no existing range resolution. Additionally, the target disturbs the reference signal, thus based on PCL principles it is difficult to detect the target at the line of sight to the transmitter as only the reference antenna is pointed in this direction, and surveillance beams are pointed in other directions. In bistatic radars a target moving at a velocity *V* provides the Doppler shift expressed as follows: [8]:

(1) $$f_{d}=\frac{2V}{\lambda }\cos\left(\alpha \right)\cos\left(\beta /2\right).$$

As previously mentioned, in PFSR $\beta \approx 180^{\circ }$, which makes $f_{d}\approx 0$ (Hz). However, observing the object in a slightly wider range, that is $\beta \in (180^{\circ }-\Delta ,180^{\circ }+\Delta )$, where Δ is a certain angle, it is possible to measure the Doppler rate (or equivalently the chirp rate (CR)). This parameter describes the kinematic properties of the measured object.

The literature describes some methods of Doppler rate estimation in PFSR. Ustalli et al. proposed in Reference [9] a four-step processing technique for the extraction of kinematic motion parameters of targets in forward scattering radar (FSR). The method is based on multiple matched filtration of the signal with simultaneous time-frequency analysis, resulting in the precise estimation of the motion parameters of a single object near the intersection of the baseline. However, a problem may be the analysis of several objects that intersect the baseline at the same time. In addition, due to multiple matched filtration and other processing operations, the complexity of the method is significant. The same authors developed this approach and described it in Reference [10]. Another solution is to use the Radon transformation to calculate the phase acceleration [11]. After transforming the signal into a two-dimensional distribution in the TF domain, the components responsible for the Doppler rate of objects are found. Analyzing the aforementioned papers, it can be noticed that the analysis in the TF domain is the proper approach to the PFSR signal’s considerations. A waveform received by the radar can be treated as a non-stationary frequency modulated signal. In the vicinity of $f_{d}\approx 0$ (Hz) the signal can be approximated as a linear frequency modulated waveform. This methodology was also presented in References [12,13,14]. This is due to the fact that the phase of the received signal can be described by the dependency:

(2) $$\phi \left(t\right)=-\frac{2\pi }{\lambda }\left[R_{1}\left(t\right)+R_{2}\left(t\right)-L\right],$$

which can be approximated using Taylor expansion into the following formula [15]:

(3) $$\phi \left(t\right)\approx \frac{\pi }{\lambda }v_{p}\left(\frac{1}{L-D}+\frac{1}{D}\right){\left(t-t_{0}\right)}^{2},$$

where $v_{p}$ is the velocity component perpendicular to the baseline *L*, $t_{0}$ is the moment when the target crosses the baseline and *D* is the range from the receiver to the crossing point (see Figure 1). As can be noted, Equation (3) describes the second order polynomial characteristic for the frequency modulated signals.

The above considerations prompted the authors to use the CR estimation in the TF domain to analyze signals from PFSR. This approach is based on short–time Fourier transform (STFT) modification and allows the CR at each point in the TF plane to be determined. This is consistent with the assumption that when the baseline and the trajectory of the object are crossed, the signal appearing in the receiver can be approximated with the linear frequency modulated waveform, and this technique is dedicated for such a problem. In addition, the method is computationally efficient, which reduces the calculation time.

This paper is organized as follows: Section 2 presents the CR estimation theory background, including Cramer–Rao lower bound (CRLB) analysis. Section 3 depicts simulation results, and Section 4 covers real-life signal analysis provided by the GSM PFSR. Discussion and comments close the article.

## 2. Chirp Rate Estimation

### 2.1. Theory Background

The pioneer of CR estimation in the TF domain using the complex phase of STFT was Czarnecki, who proposed a method for determining the instantaneous frequency rate a two-dimensional signal distribution [16,17]. In general, the signal described by the following model will be considered:

(4) $$x\left(t\right)=A_{x}\exp\left(j\Phi_{x}\left(t\right)\right),$$

for the amplitude $A_{x}$, $j=\sqrt{-1}$ and phase described as:

(5) $$\Phi_{x}\left(t\right)=\phi_{x}+\omega_{x}t+2\pi \cdot \alpha t^{2}/2=\phi_{x}+2\pi t\left(f_{0}+\alpha t/2\right),$$

where $\omega_{x}=2\pi f_{0}$ is the angular frequency with the carrier frequency $f_{0}$, and α is the CR. x(t) can be transformed into a two-dimensional distribution using STFT, which is given by the formula:

(6) $$F_{x}^{h}\left(t,\omega \right)=\int_{R}^{}x{\left(\tau \right)}^{*}h\left(t-\tau \right)e^{-j\omega \tau }d\tau ,$$

where ${\left(\cdot \right)}^{*}$ is the complex conjugate, and the upper index in the $F_{x}^{h}$ expression denotes the analysis window h(t) whereas the lower index expressed the signal under consideration x(t). Energy distribution in the TF domain can be calculated as a squared absolute value of the STFT and is called a spectrogram:

(7) $$S_{x}^{h}\left(t,\omega \right)={\left|F_{x}^{h}\left(t,\omega \right)\right|}^{2},$$

where |·| denotes the absolute value operator. STFT can be presented using the concept of a complex phase in the thought of dependency [16]:

(8) $$F_{x}^{h}\left(t,\omega \right)=\int_{R}^{}x{\left(\tau \right)}^{*}h\left(t-\tau \right)e^{-j\omega \tau }d\tau =A_{x}^{h}\left(t,\omega \right)e^{j\phi_{x}^{h}\left(t,\omega \right)}=e^{\Lambda_{x}^{h}\left(t,\omega \right)+j\phi_{x}^{h}\left(t,\omega \right)},$$

where STFT phase is described as $\phi_{x}^{h}\left(t,\omega \right)$, whereas $A_{x}^{h}\left(t,\omega \right)$ denotes STFT absolute value ($A_{x}^{h}\left(t,\omega \right)>0$). By using the property described in Reference [18], the STFT phase in Equation (8) was transformed into a complex form in which ${\mathrm{>\Lambda}}_{x}^{h}\left(t,\omega \right)=\ln\left(A_{x}^{h}\left(t,\omega \right)\right)$ then the complex phase of the STFT is defined as:

(9) $$\Psi_{x}^{h}\left(t,\omega \right)=\ln\left(F_{x}^{h}\left(t,\omega \right)\right)=\Lambda_{x}^{h}\left(t,\omega \right)+j\phi_{x}^{h}\left(t,\omega \right).$$

such a transformation allows many useful signal parameters in the TF domain to be determined. By calculating the partial derivatives of the real part of the complex phase with respect to time and frequency, the instantaneous bandwidth and the local group delay are obtained respectively. The ratio of these values gives the CR estimator in the manner described as follows:

(10) $$K\left(t,\omega \right)=-\left(\frac{\partial \Lambda_{x}^{h}\left(t,\omega \right)}{\partial t}/\frac{\partial \Lambda_{x}^{h}\left(t,\omega \right)}{\partial \omega }\right).$$

**Figure 2:**
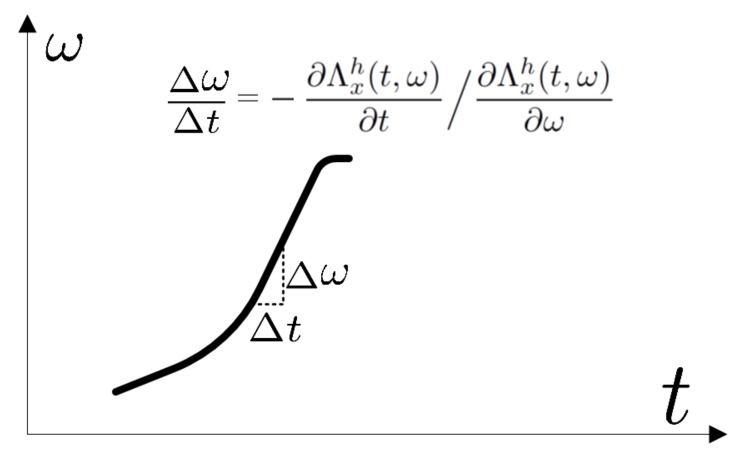
CR estimation in the TF domain—an interpretation.

The graphic interpretation of the estimator is presented in Figure 2.

In Reference [19] it was proposed that the K estimator can be calculated more efficiently utilizing the modified analysis window. Additionally, two new estimators were revealed. The uncertainty effect occurring in the K estimator has been reduced by the differentiation of the numerator and the denominator with respect to time (giving the D estimator) and frequency (giving the F estimator) giving the following relationships:

(11) $$D\left(t,\omega \right)=-\left(\frac{\partial^{2}\Lambda_{x}^{h}\left(t,\omega \right)}{\partial t^{2}}/\frac{\partial^{2}\Lambda_{x}^{h}\left(t,\omega \right)}{\partial \omega \partial t}\right),$$

(12) $$F\left(t,\omega \right)=-\left(\frac{\partial^{2}\Lambda_{x}^{h}\left(t,\omega \right)}{\partial \omega \partial t}/\frac{\partial^{2}\Lambda_{x}^{h}\left(t,\omega \right)}{\partial \omega^{2}}\right).$$

This method was tested using different types of signals and is described in the literature. Acoustic signals were processed using this method, which can be found in References [17,20]. Radar applications utilizing this approach are presented in References [21,22,23]. In this paper, the PFSR application is proposed in order to verify the possibility af assessing the motion parameters of a target. Because the estimators given by Equations (10)–(12) are actually equivalent, further considerations are made using one of them to present the correctness of the concept. This is an example of using the idea in PFSR applications, and each of the estimators should give similar results. The comparison of estimators as well as their limitations and computational complexity have been made in the literature [19,21]. Due to the smaller variance in comparison to the estimator K (see Section 2.2) and the lower sensitivity to noise (see Reference [21]), the authors decided to perform the tests using the estimator F; however, the estimators K and D can be used in the same way.

### 2.2. Analysis of the Estimation Accuracy

The accuracy of the estimators has been compared in this section to the CRLB for the considered signal model. The analyzed complex chirp signal is given by:

(13) $$x\left[n\right]=A_{x}\exp\left(j\alpha \frac{n^{2}}{2}\right),$$

where $A_{x}=1$, n∈[0,N-1]. This signal is merged in a white Gaussian noise w[n] with a variance $\sigma_{w}^{2}$. Thus, the observation vector $x={\left[x[0],x[1],\ldots ,x[N-2],x[N-1]\right]}^{T}$ is normally distributed:

(14) x∼N(μ(α),C(α)),

where

(15) $$\mu \left(\alpha \right)=x={\left[A_{x},A_{x}\exp\left(j\alpha \frac{1^{2}}{2}\right),A_{x}\exp\left(j\alpha \frac{2^{2}}{2}\right),\ldots ,A_{x}\exp\left(j\alpha \frac{{\left(N-1\right)}^{2}}{2}\right)\right]}^{T},$$

and C(α) is a covariance matrix of observation vector whereas ${\left(\cdot \right)}^{T}$ is the matrix transpose. For such a Gaussian observation model depending on the scalar parameter, α the Fisher information matrix (FIM) is a 1 by 1 matrix (scalar) given by [24]:

(16) $$I\left(\alpha \right)=2ℜ\left({\left[\frac{\partial \mu (\alpha )}{\partial \alpha }\right]}^{H}C^{-1}\left(\alpha \right)\left[\frac{\partial \mu (\alpha )}{\partial \alpha }\right]\right)+\frac{1}{2}T\left[{\left(C^{-1}\left(\alpha \right)\frac{\partial C(\alpha )}{\partial \alpha }\right)}^{2}\right],$$

where ${\left(\cdot \right)}^{H}$ is the Hermitian transpose and T denotes the matrix trace and *ℜ* expresses a real part. Since, the additive noise w[n] is white, the covariance matrix C(α) equals $\sigma_{w}^{2}I$ and does not depend on the parameter α. Thus $\frac{\partial C(\alpha )}{\partial \alpha }=0$ and the second term in Equation (15) vanishes. Moreover, $C^{-1}\left(\alpha \right)=\frac{1}{\sigma_{w}^{2}}I$. The FIM now takes the following form:

(17) $$I\left(\alpha \right)=2ℜ\left({\frac{1}{\sigma_{w}^{2}}\left[\frac{\partial \mu \left(\alpha \right)}{\partial \alpha }\right]}^{H}\left[\frac{\partial \mu \left(\alpha \right)}{\partial \alpha }\right]\right).$$

According to Equation (15) it can be written as:

(18) $$\begin{array}{c} \begin{array}{c} I\left(\alpha \right)=2ℜ\left(\frac{1}{\sigma_{w}^{2}}\sum_{n=0}^{N-1}\left(\frac{\partial }{\partial \alpha }A_{x}e^{\left(\frac{-j\alpha n^{2}}{2}\right)}\right)\left(\frac{\partial }{\partial \alpha }A_{x}e^{\left(\frac{j\alpha n^{2}}{2}\right)}\right)\right)=\\ =2ℜ\frac{A_{x}^{2}}{\sigma_{w}^{2}}\left(\sum_{n=0}^{N-1}e^{\left(\frac{-j\alpha n^{2}}{2}\right)}\left(-j\frac{n^{2}}{2}\right)e^{\left(\frac{j\alpha n^{2}}{2}\right)}\left(j\frac{n^{2}}{2}\right)\right)=\frac{A_{x}^{2}}{{2\sigma }_{w}^{2}}\sum_{n=0}^{N-1}n^{4}. \end{array} \end{array}$$

Finally, the CRLB for the estimator variance is given by:

(19) $$\sigma^{2}\left(\hat{\alpha }\right)\geq {\left[I\left(\alpha \right)\right]}^{-1}=\frac{{2\sigma }_{w}^{2}}{A_{x}^{2}\sum_{n=0}^{N-1}n^{4}}.$$

**Figure 3:**
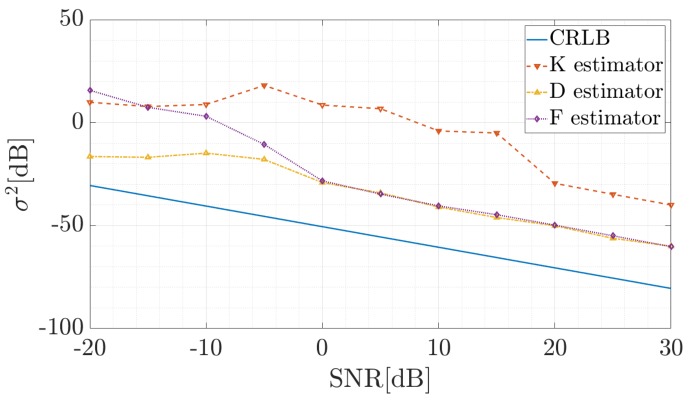
Comparison of the accuracy of the utilized estimators.

In reference to the article describing the estimators used [19], the accuracy of the estimate was investigated and compared to the presented CRLB given by Equation (19). The linear complex chirp was considered. The signal model given by Equation (13) has the following parameters: N=250, $\alpha =2\pi \frac{0.36}{N}$. In 1000 realizations of noise in the range from −20 to 30 dB, the CR value was estimated and verified at point $n_{0}$ on the reference frequency $f_{\mathrm{ref}}=\frac{\alpha_{\mathrm{ref}}}{2\pi }n_{0}$, where $\alpha_{\mathrm{ref}}=0.009$. Results for the estimators K, D and F are presented in Figure 3.

As can be seen, for signals with signal–noise ratio (SNR) ≥0 dB the D and F estimator give very similar results. The signals from this range will be considered later in the paper, so choosing one of the two more accurate tools was right. Additionally, according to the authors’ experience, this estimator is less sensitive to the changes of the analysis window width. Because of the uncertainty problem in the K estimator, the CR was verified in the vicinity of the $f_{\mathrm{ref}}$. For this reason, the variance of this tool is clearly greater than in the case of the other two estimators. Although all of the tools used are characterized by an error in relation to the CRLB, it should be kept in mind that for SNR ≥0 dB the variance of the D and F estimators is satisfactorily low, and in many practical cases is sufficient to estimate CR.

## 3. Results of the Simulation

**Figure 4:**
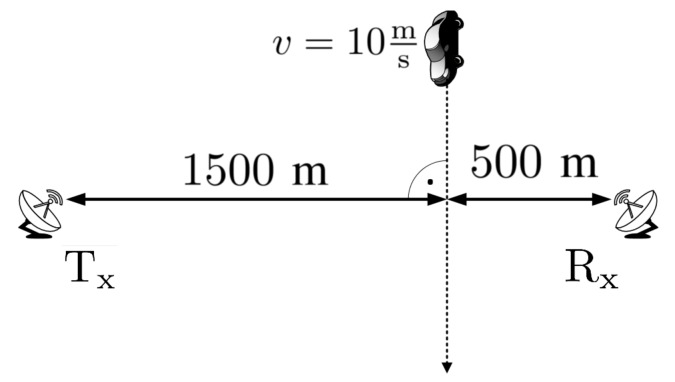
Radar scene for the 1st simulation case.

In order to verify the proposed method simulations were carried out. Two radar scenes were considered in which one or two targets crossed the baseline. The first of the analyzed cases covers the situation presented in Figure 4.

**Figure 5:**
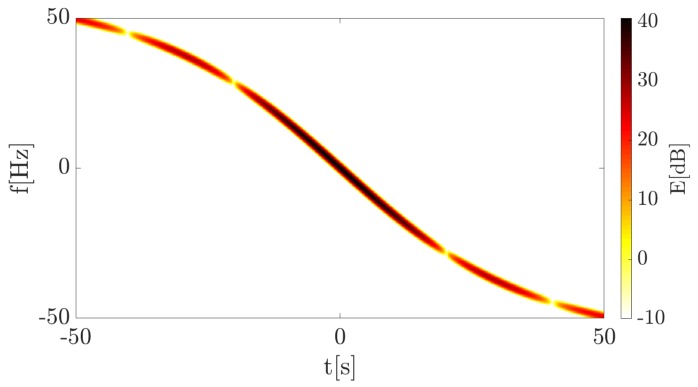
Spectrogram of the 1st simulation case.

The continuous wave (CW) transmitter working with the harmonic signal using the carrier frequency $f_{c}=900$ MHz is 1500 m away from the target trajectory (at the closest point) which, in turn, is 500 m from the receiver. The point target (dimension is neglected) moves with the velocity $v=10\frac{m}{s}$ perpendicularly to the baseline. The TF distribution of the signal energy in the form of a spectrogram is presented in Figure 5. The signal was merged with the white Gaussian noise, for which SNR = 30 dB. This was due to the fact that in the further part of the article covering the real-life signal analysis, waveforms characterized by SNR > 0 dB are considered, and on such conditions the simulations were focused. STFT parameters during the simulations are as follows:

- N = 1024—amount of points in FFT analysis,
- W = 350—Blackman-Harris window length (in samples),
- H = 1—hop-size (in samples),
- $f_{s}=1$ kHz—sampling rate.

**Figure 6:**
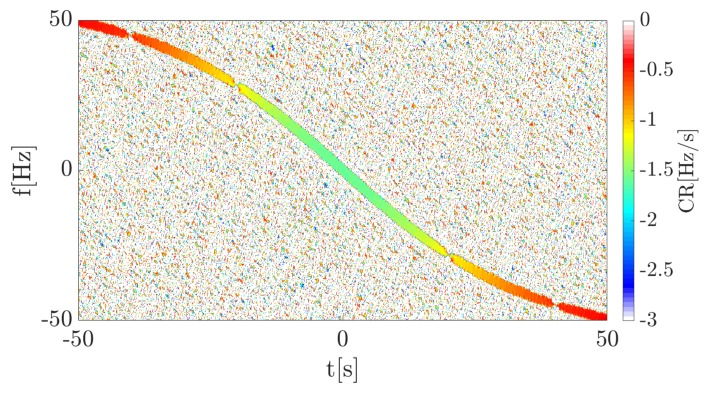
Accelerogram of the 1st simulation case.

The spectrogram shows a characteristic curve, typical for a bistatic geometry utilizing the forward scattering phenomenon. The received signal is defined only by the Doppler frequency (due to the fact that the transmitter works with the CW) expressed by Equation (1), the consequence of which is the frequency modulation of the wave. The Doppler rate, in this case, can be considered as the CR described in the previous section and the physical interpretation of both is the same. Thus, the proposed CR estimation methods were employed to verify their usability in such a context. A calculated accelerogram presenting instantaneous CR for each point in the TF plane is depicted in Figure 6.

By analyzing this distribution, it can be noted that during the crossing of the baseline the target provided a Doppler rate of ∼$-1.5\frac{m}{s}$. Additionally, the Doppler rate can be determined for each point for the observation time. This is an advantage of the proposed approach due to the fact that the estimation process automatically provides a set of information about the movement parameters of the target, allowing an unknown trajectory or velocity to be assessed. In comparison to the methods presented in the literature, unique movement signatures are provided by this approach, which allows additional information about the object to be distinguished.

**Figure 7:**
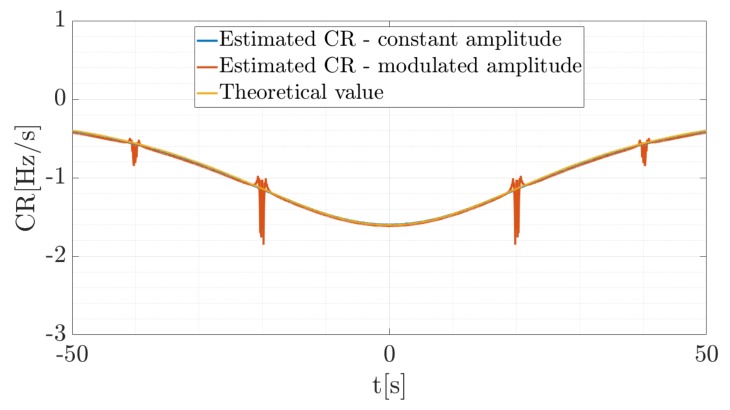
Comparison of the true Doppler rate and estimated CR for two amplitude cases.

For the purpose of validation, the theoretical Doppler rate, as the first order derivative of the $f_{d}$ given by Equation (1), was compared with the estimated value. This value was read in the maximum point of the spectrogram in each time frame. Because of the amplitude modulation, the estimated value in the minimum of the envelope is distorted, thus additionally results for the constant signal amplitude were plotted. A comparison of the true value and estimated CR for both cases (amplitude modulation and no amplitude modulation) is presented in Figure 7.

As can be noticed, the estimated Doppler rate (CR) for the constant amplitude signal is similar to the theoretical value. In Figure 7 the blue line overlapped completely with the yellow line, which confirms the convergence of the results of the estimation with the theory. However, because of the fact that the signal has an amplitude locally near or equal to zero, the estimation process returns distorted results (see the red line in Figure 7). This is caused by the character of the signal, not by the error of the estimation, which can be seen in the case where the amplitude is constant. In the simulation, the maximum value of the spectrogram in each time portion was extracted, so moments in which signal amplitude is near to zero, the maximum energy value was found in the noise, which caused significant errors.

**Figure 8:**
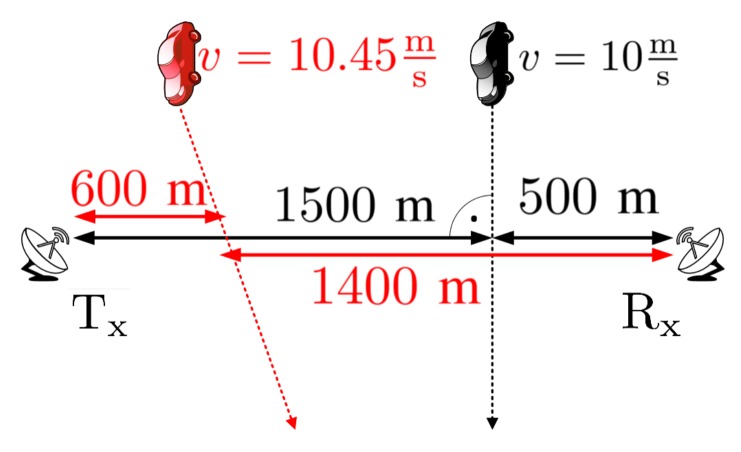
Radar scene for the 2nd simulation case.

In order to present the advantages of the proposed method, an extended scenario of the simulation was carried out. In this situation two objects are considered, however, one of them approaches the baseline from a different angle in comparison to the first one, and with slightly higher velocity. The simulation parameters are presented in Figure 8.

**Figure 9:**
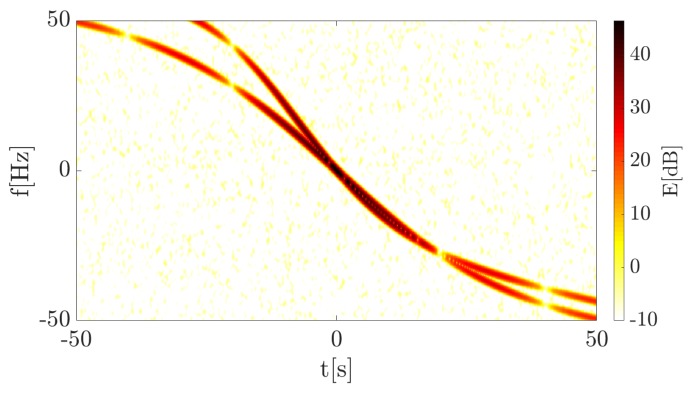
Spectrogram of the 2nd simulation case.

A spectrogram presenting the energy distribution of the reflected signal from the two targets is depicted in Figure 9. The additional target introduces a second curve with a different Doppler rate depending on the velocity and trajectory. Thus, based on the previous results it should be possible to differentiate considered targets on the accelerogram because of the individual Doppler rate seen during the time of analysis.

**Figure 10:**
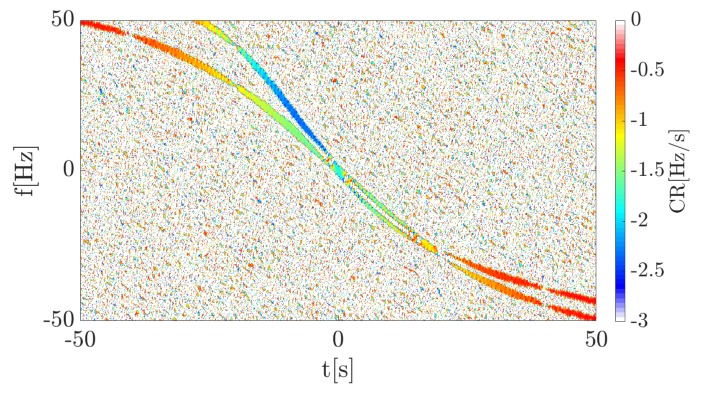
Accelerogram of the 2nd simulation case.

In the simulated case, it was assumed that both targets cross the baseline approximately the same time in order to verify the possibility of extracting particular targets in PFSR geometry. An accelerogram of the simulated data is presented in Figure 9 and Figure 10.

Based on the simulation carried out it was proved that CR estimation may be an effective technique, allowing additional information in PFSR to be extracted. Even if the Doppler rate in the vicinity of t=0 (s) is similar for both targets, the Doppler rate history from the entire observation time provides additional information about the trajectory of the objects. Such data can be used as an extension for PFSR systems. In the next section, the method is tested using real-life data to verify the adaptability and usability of the proposed approach.

## 4. Real-Life Signal Analysis

### 4.1. Measurement Campaign

The experimental data were gathered during a measurement campaign using a GSM-based passive radar for monitoring ground moving objects. The source of the illuminating signal was a GSM base transceiver station with an antenna mounted at the height of approximately 50 m. During the trials, a cooperating vehicle was used as an observed target moving with the velocity $v\approx 10\frac{m}{s}$ [25]. Measurements were carried out a few times under similar conditions. This was done to confirm the repeatability of results in comparable scenarios, and to verify the possibility of distinguishing characteristic features in the movement.

The location of the radar’s surveillance antenna and trajectory of the target were selected in order to ensure that the forward scattering effect of the vehicle occurred at the observation spot. The surveillance antenna was mounted 1.5 m above the ground and was located approximately 1.5 km from the transmitter. The cooperating car crossed the baseline between the transmitter and the receiver within approximately 1 km of the transmitter.

The reference signal was acquired at the same location as where the measurement was performed. In order to reduce the influence of the target echo in the reference channel, an antenna with vertical beamwidth of approximately 25 degrees was mounted on a 12 m mast and tilted upwards.

**Figure 11:**
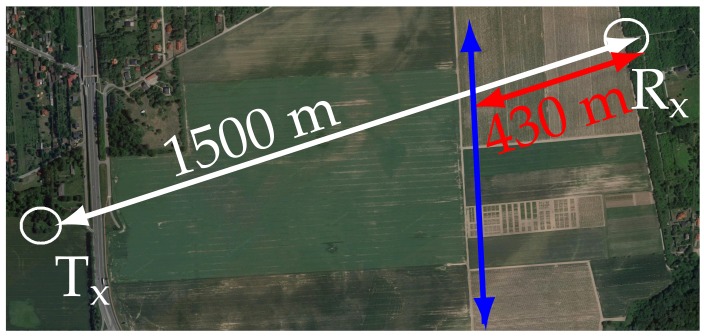
Measurement scene. In white—Range from the GSM transmitter to the receiver, in red—range from the receiver to the intersection point, in blue—the target trajectory.

**Figure 12:**
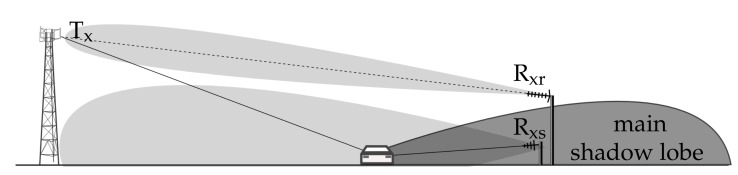
Measurement geometry diagram. $T_{x}$—GSM transmitter of opportunity, $R_{\mathrm{xr}}$—the reference antenna, $R_{\mathrm{xs}}$—the surveillance antenna.

The measurement equipment consisted of commercial-off-the shelf components, with a two channel receiver based on the National Instruments PXIe-5667 vector signal analyzer, low noise amplifiers and band-pass filters for the GSM900 band (925–960 MHz) connected after the antennas of both the surveillance and reference channels. The measurement scene utilized during the trials is presented in Figure 11, and the general measurement geometry diagram is depicted in Figure 12. More details about the measurement campaign and the signal processing chain are available in Reference [25].

### 4.2. Target Doppler Rate Estimation

The obtained data were examined with one of the analyzed estimators in order to verify whether the proposed tool is effective in analyzing real-life signals from PFSR. The recorded waveform was processed using the cross-correlation of the reference and reflected from the target signal. As with the simulation, the object’s dimensions can be neglected. This is due to the fact that the available GSM signal bandwidth is ∼200 kHz, giving a bistatic range resolution of ∼1500 m. Four cases were considered during which the object examined crossed the baseline between the transmitter and the receiver. During the real-life signal analysis, the following parameters were employed:

- N = 4096—amount of points in FFT analysis,
- W = 1300—Blackman-Harris window length (in samples),
- H = 1—hop-size (in samples),
- $f_{s}=1$ kHz—sampling rate (after decimation).

**Figure 13:**
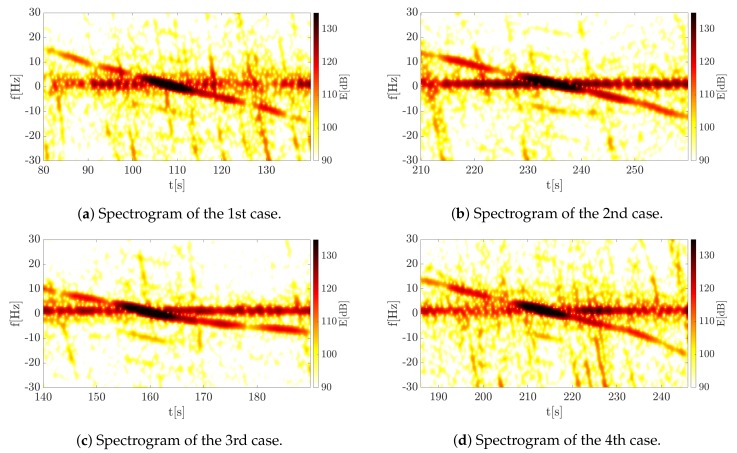
Spectrograms of all considered cases.

The spectrograms of the analyzed cases are presented in Figure 13.

Apart from the signal that is caused by the analyzed phenomenon, that is the signal with frequency modulation, additional components are visible on the spectrograms. They arise for several reasons. The first of these is the presence of a significant number of stationary objects in the radar beam. A strong echo from the surface of the earth, trees or buildings creates a strong permanent component at f≈0 (Hz), the impact of which can be reduced, as described in Reference [25], for example. The second effect is visible as numerous components with a much higher modulation coefficient. However, these are only visible for a short time. This phenomenon, in turn, arises as a result of the presence of side lobes, which cause the receiving of a signal reflected from a moving object as well as from other objects in space. In addition, there was a highway near the measuring scene, and the cars moving on it are visible in the spectrograms. However, it is possible to distinguish a significant component, which is the echo coming from the analyzed object.

**Figure 14:**
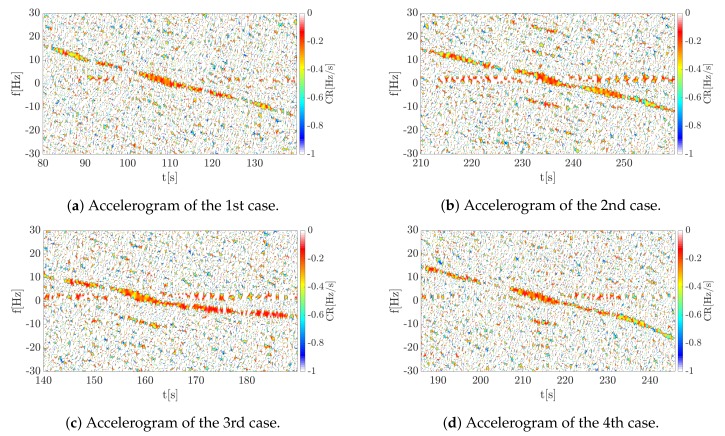
Accelerograms of all considered cases.

For such obtained data, accelerograms, that is a local (instantaneous) CR distribution on the TF plane, were determined. The results for the analyzed cases are shown in Figure 14.

As can be noticed, similar results were obtained for four measurements which confirmed the repeatability of the results. Although the most significant portion of the signal energy is at the moment of changing the frequency sign (around 0 Hz), it is possible, as in the case of the simulation, to determine the CR for the entire recorded segment. Such information can be extremely useful when determining the trajectory or kinematic parameters of the target. In the case of the previously mentioned solutions [9,10,11,12,13,14,15], only the Doppler rate was determined at the moment when the baseline is crossed. The proposed method therefore extends the spectrum of possibilities, allowing more information to be extracted. For all cases the estimated CR value at the time of $t_{0}$ is CR ∈[-0.3,-0.5] (Hz/s), which agrees with the actual value. For example in the first case shown in Figure 13a and Figure 14a, it can be observed that the frequency is reduced by less than 30 Hz within 60 s, which gives an estimated value. In fact, it is difficult to reproduce the vehicle’s motion perfectly a few times, which resulted in temporary changes in the signal. It can be observed by comparing the 1st and 2nd cases (corresponding spectrograms Figure 13a,b and accelerograms Figure 14a,b) with 3rd and 4th (corresponding spectrograms Figure 13c,d and accelerograms Figure 14c,d). For the first two cases the car maintains a constant velocity, while for the other two cases the velocity is not retained in the second phase of the move. For the third case it is noticeable that the car moves slowly, which results in a decrease in the chirp rate (absolute) value. In the fourth case, the acceleration of the car is noticeable, which was also observed. In this situation, the accelerogram presents more rapid changes in the range of -0.5∼-0.8$\frac{\mathrm{Hz}}{s}$ in the final part of the observed movement. The presented results confirmed the correctness of the proposed method in the estimation of the object motion parameters observed by the PFSR radar, as well as extraction characteristic features of the object movement. The tested algorithms can help and/or speed up the estimation of the object’s parameters, which is especially important due to the fact that information about the distance of the object is lost in the PFSR radar. In this situation, any additional information about the object may be useful.

## 5. Conclusions

The article has presented a novel approach to the analysis of signals occurring in radars using the forward scattering phenomenon. Based on the concept of the complex STFT phase and the CR estimators known from the literature, the signals from the PFSR radar have been analyzed. In the first part, using a mathematical model, the possibility of applying the proposed method has been tested using simulated signals. A situation has been considered in which one object and two objects with different kinematic parameters intersect the baseline. Simulations have confirmed the applicability of the method, after which the method was verified using real-life data. The role of the illuminator of opportunity has been fulfilled by the GSM transmitter and the transmitter-receiver line was crossed by the car. The analysis allowed the Doppler rate to be determined in the real scenario. In addition, the method gives the Doppler rate estimation, which had not yet been available, not only at the time of the baseline intersection, but also for the entire observed trajectory. A valuable property is the ability to extract temporary changes in velocity, which increases the amount of information describing the observed object.

The accuracy of the considered tools has been verified by statistical analysis and the comparison of results to the CRLB, which mainly showed the differences between the estimators as well as the expected accuracy. In the future, the authors want to verify the accuracy of the estimators for a different analysis window length. This is an important parameter due to the fact that in the classical STFT the length of the analysis window affects the estimation variance and bias, and because the tools used are based on STFT, the bias and the variance are also related to the analysis window’s length.

A promising point of further work is the extension of the method with the possibility of classifying objects and estimating the targets tracks. Based on the Doppler rate, not only at the intersection of the baseline but also in the wider observation period, it is possible to classify objects. This can be particularly important in security systems, where the detection of fast and maneuvering missiles is difficult for both active and passive radars. The use of the proposed approach for a transmitter located on the ground and a receiver on the ground or in orbit would allow for detection and classification of dangerous rockets and flying objects.
